# Acute and Long-Term Toxicity of Mango Leaves Extract in Mice and Rats

**DOI:** 10.1155/2014/691574

**Published:** 2014-08-27

**Authors:** Yi Zhang, Jian Li, Zhizhen Wu, Erwei Liu, Pingping Shi, Lifeng Han, Lingling Guo, Xiumei Gao, Tao Wang

**Affiliations:** ^1^Tianjin State Key Laboratory of Modern Chinese Medicine, 312 Anshanxi Road, Nankai District, Tianjin 300193, China; ^2^Institute of Traditional Chinese Medicine, Tianjin University of Traditional Chinese Medicine, 312 Anshanxi Road, Nankai District, Tianjin 300193, China; ^3^Key Laboratory of Pharmacology of Traditional Chinese Medical Formulae, Tianjin University of Traditional Chinese Medicine, Ministry of Education, 312 Anshanxi Road, Nankai District, Tianjin 300193, China

## Abstract

The acute toxicity of mango leaves extract (MLE) at the maximal dose (18.4 g/kg) was studied in ICR mice and no abnormalities were detected during the experiment. The long-term studies at various doses of MLE (100 mg/kg, 300 mg/kg, and 900 mg/kg) in SD rats for 3 consecutive months revealed that, compared with the control group, rats in MLE treated groups showed slight body weight increase and higher fat weight; the serum TG and CHOL levels and the epididymis weight of male rats were a little higher; the serum K^+^ level of female rats was on the low side but the weights of liver, kidney, and adrenal gland were on the high side. In addition to this, no other obvious abnormalities were detected.

## 1. Introduction

Mango tree (*Mangifera indica *L.), a tropical plant belonging to Anacardiaceae, has been distributed worldwide as the most cultivated fruits in the tropics. Mango leaves were used for diabetes and asthma treatment in traditional Chinese medicine (TCM). Mango leaves contain phenolic constituents such as caffeic acid [1], polyphenols such as mangiferin and gallic acid [2], flavonoids [3], volatile compounds [4], and so forth. Pharmacology studies showed that the extract of mango leaves possesses many effects like antioxidant, antimicrobial, antihelminthic, antidiabetic, antiallergic, and so forth [5]. In previous study, we reported benzophenone C-glycosides with triglyceride accumulation inhibitory effects in adipocyte [6, 7]. We also reported that ethanol extract of mango leaves dose-dependently decreased serum glucose and triglyceride in KK-A^y^ mice, and mechanism on glucose and lipid homeostasis is mediated, at least in part, through PI3K/AKT and AMPK signaling pathway [8]. Although mango tree leaves were used for a long period in TCM clinic, there are few reports on the safety evaluations. In this study, we carried out the acute toxicity and long-term toxicity of mango leaves extract (MLE), aiming at providing reference basis for other safety evaluation studies and selecting clinical dosage.

## 2. Materials and Methods

### 2.1. Materials

#### 2.1.1. Plant Material

In the present study, mango leaves were collected from Zhejiang Province, China, and identified by Dr. Tianxiang Li at Tianjin University of TCM as* Mangifera indica* L. Voucher specimen was deposited at the Academy of Traditional Chinese Medicine of Tianjin University of TCM

#### 2.1.2. Animals

Forty ICR mice, half male and half female, weighted 18–21 g, and 48 SD rats, composed of male and female in half, weighted 117–160 g, were used in the study and feed in room of SPF grade laboratory. These animals were all provided by Vital River Laboratory Animal Technology Co., Ltd.

#### 2.1.3. Instruments

PL203 electronic balance and ML203 electronic balance were purchased from Changzhou Mettler Toledo Instrument Co., Ltd. ADVIA2120 hematology analyzer was made by Germany Siemens Electrical Apparatus Ltd. ACL9000 coagulometer was made by American Beckman Coulter Inc. 7080 automatic biochemical analyzer was made by Japanese Hitachi Ltd.

### 2.2. Methods

#### 2.2.1. Preparation of MLE

Dried mango leaves, collected from Hainan province of China, was extracted by 70% ethanol (1 g in 10 mL) under reflux for 2 h, and the residue was extracted under the same condition. The 70% ethanol solutions were combined together and further subjected to a D101 macroporous absorption resin column eluted with water, 15% ethanol. 15% ethanol fraction was concentrated and reextracted by 50% ethanol (50 degrees for 2 h). The 50% ethanol extract was dried under vacuum to obtain mango leaves extract, which contains 62% mangiferin (HPLC method). The final extract based on the above process was effective and has the functions of mango leaves.

#### 2.2.2. Acute Toxicity of MLE in ICR Mice

Forty ICR mice of mixed sexes were randomly divided into two groups: MLE treated group and control group. Mice in MLE treated group were given MLE at the maximal dose of 18.4 g/kg by intragastric administration, in a volume of 0.1 mL per 10 g, twice a day with a 4 h interval, while the control group received an equal volume of deionized water. After oral administration, the various responses of mice including toxic reactions and mortality were observed and recorded every day for successive 14 days. At the end of the experiment, animals were executed for gross anatomy check. Evaluating and recording whether there were any obvious changes in major organs by macroscopic observation.

#### 2.2.3. Long-Term Toxicity of MLE in SD Rats

*(1) Group Setup and Administration.* After seven days of stabilization, 48 SD rats of mixed sexes that have moderate body weight and body weight gain speed were selected for the study. Depending on the weight, they were randomly divided into four groups, including one blank group for control and three administration groups of different doses (100 mg/kg, 300 mg/kg, and 900 mg/kg) which are equivalent to 17.2, 51.7, and 155.2 times of clinical daily dose (5.8 mg/kg), respectively. Rats in MLE treated group were given MLE by intragastric administration at the corresponding dose, in a volume of 0.1 mL per 10 g, for 3 consecutive months (6 times a week), while the control group received an equal volume of deionized water. During the study, all rats were allowed access to food and water ad libitum.

*(2) Observational Indices.* After oral administration, observe the general symptom, such as appearance, behavior, glandular secretion, breathing, and so on. The body weight and food consumption of each animal were recorded weekly and the differences among groups were compared.

After 90 days of treatment, all the 48 SD rats were sacrificed, and blood samples were collected from the abdominal aorta for hematology and coagulation tests. The white blood corpuscles (WBC) count, red blood corpuscles (RBC) count, hemoglobin concentration (HGB), hematocrit (HCT), mean corpuscular volume (MCV), and prothrombin time (PT) were carried out.

Serum was separated by spinning the blood and was used for biochemical studies. ALT, AST, ALP, BUN, CREA, TP, ALB, GLU, CHOL, Na^+^, K^+^, and Cl^−^ contents in serum and many other blood biochemical parameters were determined using the automatic biochemistry analyzer.

Organs (liver, heart, spleen, lung, kidney, brain, thymus, etc.) were collected from each sacrificed rat and weighed. The relative organ weights (organ/body weight ratio and organ/brain weight ratio) were calculated and compared with the value of the control.

#### 2.2.4. Statistical Analysis

The intragroup difference of measurement data was detected with the *t*-test. The data obtained were subjected to SPSS NPar Tests Mann-Whitney Test. Values were expressed as the mean ± standard error and were considered statistically significant at *P* < 0.05.

## 3. Results and Discussion

### 3.1. Acute Toxicity

#### 3.1.1. General Observation

During the course of the study, all the mice were healthy without any abnormal responses, and no distinct lesions were revealed anatomically.

#### 3.1.2. Body Weight

After the second oral administration of MLE, the body weight of the mice in both of the two groups decreased slightly than before the first dose, and the control group decreased more, but there was no significant difference (*P* > 0.05). It is speculated that the body weight loss may result from the fasting between the two doses and the high drug concentration (the maximum dispensing concentration) that induced satiety may affect the short-time body weight change.

Throughout the recovery period, the animal weight, both of the two groups, showed a general increase, but in several mice it decreased a day after the treatment (one in control group and six in MLE treated group); and after fourteen-day treatment, five mice in MLE treated group showed a slight weight loss (less than 1 g). Speculated by the whole growth trend, we guess that the body weight change after one-day treatment, which was recovered three days later, may be associated with the drug's effect and the change after fourteen days was likely to be coursed by physiological fluctuations, but it produced no significant difference (*P* > 0.05) compared with control group (Table 1). As a result, the effect of MLE on mice body weight was not obvious.

#### 3.1.3. Gross Anatomy and Histopathological Examination

After a fourteen-day recovery, all the mice were executed for gross anatomy check. Because there were no gross lesions on major organs, no histopathological examination was conducted.

### 3.2. Long-Term Toxicity

#### 3.2.1. General Observation

After three consecutive months of oral administration, all the animals showed no marked abnormalities during the study.

#### 3.2.2. Body Weight and Food Consumption

The body weight of rats (presented in Table 2) in each group showed a steady increase trend, while MLE treated groups had a higher body-mass index than control group. However, there were no significant differences (*P* > 0.05), except in the female rats in low and medium dose groups at day 10 and in high dose group at days 21 and 49 (*P* < 0.05).

The food consumption of rats (presented in Table 3) in each group showed some fluctuation, but there were no significant differences among those groups (*P* > 0.05).

As a result, after a long-term administration of MLE, the effects on the body weight and food consumption of rats were not obvious.

#### 3.2.3. Hematology and Coagulation Function

After a 3-month of oral administration of MLE, some hematology indices and coagulation function of rats were determined as were shown in Table 4.

Compared with control group, the mean corpuscular volume (MCV) of male rats in low and medium dose groups and the mean corpuscular hemoglobin (MCH) of male rats in medium dose group were significantly decreased (*P* < 0.05 or *P* < 0.01). The RBC, HGB, and HCT in serum, however, were normal and the change extents of MCV and MCH were in a narrow range and no abnormalities were found in high dose group but the RBC was significantly elevated (*P* < 0.05).

Prothrombin times of rats in each group were approximately the same (*P* > 0.05) and were in normal range.

As a result, 3-month consecutive oral administration of MLE caused no obvious influences to the hematology and coagulation function of rats.

#### 3.2.4. Biochemical Parameters

Serum levels of TG and CHOL of the male rats in high dose group were significantly higher (*P* < 0.05) than that of control group. Among the female rats, the AST and TBIL levels in high dose group were significantly lower (*P* < 0.05) but within a narrow range than the controls, which means no toxicological significance. Moreover, a lower serum K^+^ level was detected in female rats in high dose group (*P* < 0.05); in addition, no other biochemical parameters were found abnormal (Table 5).

#### 3.2.5. Relative Organ Weight

The effects of MLE on the relative organ weights of male rats are presented in Table 6. Compared with control group, epididymis weight in medium and high dose groups were significantly higher (*P* < 0.05 or *P* < 0.01) and epididymis/brain weight ratio in high dose group was significantly different (*P* < 0.05), while the organ/body weight ratio revealed no marked differences (*P* > 0.05). The fat weight and the relative fat weight were higher but produced no remarkable differences than the controls (*P* > 0.05) and there were no obvious changes with the increasing of the dosage of MLE. Besides, all the other organ weights were normal.

The effects of MLE on the relative organ weights of female rats are presented in Table 7. Compared with control group, the liver/body weight ratio and the liver/brain weight ratio in medium dose group were remarkably different (*P* < 0.05), and in the high dose group, the kidney weight, the relative kidney weights, the adrenal gland weight, and the ratio of adrenal gland to brain weight were also significantly different (*P* < 0.05 or *P* < 0.01). While in the low and medium groups, the fat weight and the relative fat weights were higher but produced no remarkable differences than the controls (*P* > 0.05) and there were no obvious changes with the increasing of the dosage of MLE. All the other organ weights were normal.

## 4. Conclusions

The acute toxicity study showed that MLE was safe at the maximum dose (18.4 g/kg) on ICR mice; animals behaved normally during the experiment and no gross lesions on major organs were examined.

Throughout the 3 consecutive months of oral administration of MLE at different doses (100 mg/kg, 300 mg/kg, and 900 mg/kg), rats in each group were normal in body weight and food consumption and various tests showed that higher serum TG and CHOL levels were found in male rats; lower serum K^+^ level was detected in female rats; the epididymis weight of male rats and the liver, kidney, and adrenal gland weights of female rats were found higher. In addition to this, all animals were normal in hematology, coagulation function, biochemical criteria, gross anatomy, and relative organs weight.

From the foregoing, in male rats, as the body and fat weight increased, the TG and CHOL levels increased; more attentions should be paid to body fat examination. Although the liver and kidney weights of rats were changed, no blood biochemistry changes were hence brought about.

## Figures and Tables

**Table 1:** The effect on body weight of MLE in ICR mice ($\overset{-}{X}\pm \text{SD}$, g).

Sex	Groups	Preceding the first dose	Preceding the second dose	After administration			
				Day 1	Day 3	Day 7	Day 14
♂	Control	20.84 ± 0.42	20.06 ± 0.40	21.72 ± 0.78	24.70 ± 0.81	28.06 ± 0.92	30.91 ± 1.02
	Drug	20.83 ± 0.71	20.31 ± 0.69	20.98 ± 1.28	24.13 ± 1.37	27.21 ± 1.52	29.94 ± 1.27

♀	Control	19.27 ± 0.64	18.59 ± 0.59	19.42 ± 1.27	21.04 ± 1.36	22.99 ± 0.88	25.33 ± 1.05
	Drug	19.19 ± 0.73	18.81 ± 0.84	18.99 ± 0.97	20.99 ± 0.86	23.42 ± 1.31	24.42 ± 1.28

Ten mice in each group; no difference was considered to be significant between the two groups.

**Table 2:** The effect on body weight of MLE in SD rats ($\overset{-}{X}\pm \text{SD}$, g).

Sex	Time	*n*	Control group	*n*	Low dose group	*n*	Medium dose group	*n*	High dose group
♂	0 d	18	149.1 ± 8.2	18	148.2 ± 8.6	18	147.6 ± 8.3	18	147.9 ± 7.9
	3 d	18	174.8 ± 10.9	18	172.3 ± 10.1	18	172.2 ± 10.1	18	169.6 ± 11.8
	7 d	18	206.4 ± 14.1	18	200.0 ± 14.5	18	201.0 ± 13.6	18	202.9 ± 15.9
	10 d	18	231.2 ± 17.0	18	227.7 ± 16.2	18	226.1 ± 15.3	18	227.5 ± 18.6
	14 d	18	261.4 ± 20.2	18	260.6 ± 19.9	18	257.8 ± 18.7	18	259.8 ± 22.1
	17 d	18	285.9 ± 22.8	18	284.4 ± 23.3	18	283.8 ± 21.2	18	285.3 ± 22.0
	21 d	18	311.3 ± 24.1	18	312.2 ± 28.4	18	313.2 ± 23.6	18	316.0 ± 23.9
	24 d	18	325.9 ± 26.6	18	331.4 ± 30.7	18	332.2 ± 25.0	18	336.4 ± 24.9
	28 d	18	347.6 ± 31.4	18	353.6 ± 35.7	18	350.0 ± 25.9	18	359.4 ± 27.5
	31 d	18	363.4 ± 34.9	18	370.1 ± 38.1	18	368.6 ± 28.1	18	377.1 ± 29.5
	35 d	18	379.2 ± 38.2	18	388.1 ± 40.6	18	387.1 ± 31.6	18	396.2 ± 30.3
	42 d	18	409.9 ± 44.1	18	418.0 ± 45.7	18	416.6 ± 34.7	18	425.0 ± 34.1
	49 d	18	435.1 ± 46.5	18	443.2 ± 47.5	18	439.4 ± 35.4	18	448.4 ± 35.2
	56 d	18	455.7 ± 51.1	18	465.7 ± 49.8	18	461.1 ± 38.3	18	471.5 ± 37.4
	63 d	18	482.6 ± 51.2	18	493.8 ± 51.8	18	487.8 ± 40.8	18	498.1 ± 39.6
	70 d	18	500.2 ± 51.8	18	508.7 ± 54.0	18	497.3 ± 44.6	18	516.4 ± 43.2
	77 d	18	516.8 ± 53.5	18	525.1 ± 57.8	18	517.6 ± 45.6	18	531.8 ± 44.9
	84 d	18	535.9 ± 56.1	18	538.2 ± 60.7	18	532.8 ± 47.5	18	546.9 ± 47.4

♀	0 d	18	132.4 ± 6.7	18	134.1 ± 7.3	18	133.0 ± 7.2	18	131.2 ± 7.4
	3 d	18	146.1 ± 7.7	18	149.4 ± 7.9	18	148.2 ± 9.0	18	147.8 ± 9.9
	7 d	18	159.3 ± 8.2	18	163.4 ± 8.7	18	165.1 ± 12.5	18	163.9 ± 13.1
	10 d	18	168.2 ± 9.3	18	176.4 ± 9.0*	18	176.3 ± 12.6*	18	174.8 ± 14.6
	14 d	18	181.5 ± 11.3	18	186.3 ± 9.6	18	186.9 ± 15.2	18	188.1 ± 15.3
	17 d	18	194.3 ± 11.4	18	197.0 ± 11.9	18	199.2 ± 17.6	18	202.6 ± 18.7
	21 d	18	204.3 ± 13.4	18	207.8 ± 12.0	18	211.1 ± 20.9	18	216.8 ± 21.3*
	24 d	18	212.6 ± 16.3	18	215.1 ± 13.0	18	222.1 ± 19.8	18	224.0 ± 24.7
	28 d	18	221.4 ± 18.4	18	223.1 ± 14.8	18	232.0 ± 21.9	18	233.8 ± 25.1
	31 d	18	229.7 ± 16.9	18	232.3 ± 13.0	18	239.9 ± 23.7	18	243.2 ± 24.7
	35 d	18	237.2 ± 17.5	18	241.8 ± 14.3	18	248.7 ± 25.3	18	250.4 ± 23.6
	42 d	18	250.4 ± 22.2	18	255.6 ± 15.0	18	262.2 ± 27.5	18	265.7 ± 24.5
	49 d	18	258.9 ± 18.2	18	263.6 ± 17.3	18	272.9 ± 29.3	18	274.6 ± 24.1*
	56 d	18	265.7 ± 18.3	18	267.8 ± 17.5	18	280.1 ± 26.5	18	276.8 ± 26.8
	63 d	18	279.2 ± 20.2	18	284.2 ± 16.8	18	292.8 ± 29.6	18	290.1 ± 26.8
	70 d	18	284.6 ± 21.1	18	290.4 ± 14.5	18	296.5 ± 30.3	18	295.1 ± 29.1
	77 d	18	290.0 ± 19.8	18	294.4 ± 14.4	18	302.6 ± 33.4	18	303.3 ± 26.8
	84 d	18	295.4 ± 19.1	18	300.6 ± 14.3	18	306.1 ± 29.6	18	308.4 ± 28.8

∗Means *P* < 0.05; *n* means the number of animals.

**Table 3:** The effect on food consumption of MLE in SD rats ($\overset{-}{X}\pm \text{SD}$, g).

Sex	Time	Cage number	*n*	Control group	*n*	Low dose group	*n*	Medium dose group	*n*	High dose group
♂	0 d	3	18	22.7 ± 0.9	18	23.1 ± 0.3	18	23.4 ± 0.8	18	22.4 ± 2.5
	7 d	3	18	25.5 ± 2.4	18	26.2 ± 1.1	18	26.5 ± 0.7	18	25.6 ± 0.8
	14 d	3	18	28.1 ± 1.4	18	28.4 ± 0.2	18	27.9 ± 1.3	18	27.9 ± 1.5
	21 d	3	18	28.4 ± 2.2	18	31.2 ± 1.5	18	28.8 ± 2.9	18	30.6 ± 1.0
	28 d	3	18	28.8 ± 2.9	18	30.6 ± 1.2	18	30.7 ± 0.4	18	31.9 ± 0.2
	35 d	3	18	29.7 ± 2.3	18	30.9 ± 2.1	18	31.6 ± 0.4	18	30.7 ± 1.2
	42 d	3	18	30.1 ± 2.3	18	29.6 ± 1.3	18	30.4 ± 0.7	18	29.6 ± 1.4
	49 d	5	18	32.4 ± 3.7	18	31.5 ± 1.1	18	30.6 ± 1.5	18	30.6 ± 3.0
	56 d	5	18	32.5 ± 2.0	18	33.4 ± 1.1	18	30.7 ± 0.7	18	31.3 ± 2.2
	63 d	5	18	32.7 ± 1.8	18	33.2 ± 2.3	18	32.6 ± 1.3	18	34.6 ± 2.2
	70 d	5	18	33.7 ± 3.4	18	33.6 ± 2.1	18	33.7 ± 1.8	18	33.1 ± 2.4
	77 d	5	18	32.3 ± 2.6	18	32.6 ± 4.0	18	32.4 ± 2.3	18	33.7 ± 2.4
	84 d	5	18	30.7 ± 3.1	18	31.1 ± 1.2	18	29.8 ± 1.2	18	30.7 ± 2.1

♀	0 d	3	18	17.6 ± 0.8	18	18.1 ± 0.5	18	18.8 ± 1.5	18	18.6 ± 0.9
	7 d	3	18	18.9 ± 1.2	18	17.3 ± 0.6	18	19.7 ± 1.5	18	17.9 ± 0.8
	14 d	3	18	20.0 ± 1.8	18	18.9 ± 0.5	18	21.4 ± 1.4	18	20.8 ± 1.1
	21 d	3	18	21.9 ± 2.4	18	22.1 ± 1.1	18	22.4 ± 3.2	18	21.4 ± 2.3
	28 d	3	18	19.4 ± 3.6	18	22.1 ± 1.2	18	22.5 ± 1.5	18	22.7 ± 1.2
	35 d	3	18	21.3 ± 0.4	18	21.1 ± 1.2	18	22.6 ± 1.7	18	23.1 ± 2.0
	42 d	3	18	22.2 ± 1.2	18	22.8 ± 1.9	18	23.4 ± 1.7	18	24.3 ± 1.0
	49 d	3	18	22.5 ± 2.4	18	24.6 ± 1.4	18	23.2 ± 3.0	18	22.1 ± 0.8
	56 d	3	18	21.7 ± 1.0	18	22.1 ± 1.1	18	22.8 ± 2.1	18	21.6 ± 0.4
	63 d	3	18	22.0 ± 1.3	18	22.3 ± 1.0	18	22.2 ± 2.2	18	20.8 ± 1.3
	70 d	3	18	23.9 ± 1.8	18	23.6 ± 0.9	18	23.8 ± 2.5	18	22.7 ± 1.3
	77 d	3	18	21.8 ± 2.0	18	23.8 ± 1.4	18	22.4 ± 2.8	18	21.1 ± 1.4
	84 d	3	18	20.3 ± 1.1	18	21.9 ± 0.6	18	21.2 ± 1.3	18	21.5 ± 1.9

*n* means the number of animals.

**Table 4:** The effects on hematology and coagulation function of MLE in SD rats ($\overset{-}{X}\pm \text{SD}$, g).

Sex	Parameters	Control group (*n* = 6)	Low dose group (*n* = 6)	Medium dose group (*n* = 6)	High dose group (*n* = 6)
♂	WBC (×10^9^/L)	10.08 ± 1.29	12.47 ± 3.03	11.66 ± 2.32	11.58 ± 3.36
	Neut (%)	12.06 ± 1.38	15.48 ± 4.58	12.33 ± 2.89	12.30 ± 5.21
	Lymph (%)	83.32 ± 1.64	79.10 ± 4.80	81.78 ± 3.14	81.85 ± 5.50
	Mono (%)	2.58 ± 0.64	3.02 ± 0.66	2.95 ± 0.71	3.00 ± 0.87
	Eos (%)	1.24 ± 0.33	1.10 ± 0.18	1.33 ± 0.56	1.65 ± 0.32
	RBC (×10^12^/L)	9.24 ± 0.36	9.52 ± 0.59	9.57 ± 0.36	9.79 ± 0.34∗
	HGB (g/L)	167.40 ± 6.80	166.17 ± 9.33	162.33 ± 4.27	167.50 ± 3.15
	HCT (%)	48.32 ± 1.57	48.40 ± 3.48	48.00 ± 1.08	49.87 ± 1.64
	MCV (fL)	52.28 ± 0.55	50.82 ± 0.77∗∗	50.20 ± 1.44∗	50.98 ± 2.29
	MCH (pg)	18.16 ± 1.10	17.50 ± 0.67	16.98 ± 0.57∗	17.13 ± 0.72
	MCHC (g/L)	347.20 ± 20.66	344.50 ± 13.40	338.33 ± 3.33	336.67 ± 5.99
	PLT (×10^9^/L)	1145.00 ± 121.6	1208.00 ± 158.73	1216.17 ± 98.95	1217.00 ± 104.08
	Retic (‰)	2.96 ± 0.42	2.80 ± 0.43	2.96 ± 0.36	2.56 ± 0.40
	PT (s)	16.04 ± 1.13	16.48 ± 2.71	15.68 ± 0.93	16.15 ± 1.22

♀	WBC (×10^9^/L)	5.10 ± 1.44	5.97 ± 0.89	5.77 ± 1.01	5.45 ± 1.41
	Neut (%)	15.92 ± 2.58	15.62 ± 3.79	17.77 ± 2.32	16.38 ± 5.62
	Lymph (%)	78.27 ± 2.64	78.38 ± 4.06	76.48 ± 2.70	78.08 ± 7.14
	Mono (%)	2.57 ± 0.46	3.02 ± 0.58	2.95 ± 1.11	2.73 ± 1.18
	Eos (%)	2.00 ± 0.40	1.60 ± 0.67	1.73 ± 0.29	1.97 ± 0.58
	RBC (×10^12^/L)	8.63 ± 0.23	8.81 ± 0.56	8.55 ± 0.26	8.64 ± 0.48
	HGB (g/L)	159.17 ± 2.93	158.50 ± 7.34	157.50 ± 2.88	159.17 ± 6.55
	HCT (%)	46.53 ± 1.29	46.52 ± 2.61	46.28 ± 1.16	46.45 ± 2.14
	MCV (fL)	53.92 ± 1.66	52.83 ± 1.26	54.18 ± 1.70	53.78 ± 0.95
	MCH (pg)	18.43 ± 0.46	18.05 ± 0.63	18.43 ± 0.37	18.47 ± 0.32
	MCHC (g/L)	342.17 ± 5.78	341.33 ± 5.79	340.50 ± 6.06	343.50 ± 2.88
	PLT (×10^9^/L)	1134.67 ± 63.64	1268.33 ± 182.79	1211.17 ± 121.14	1197.83 ± 73.19
	Retic (‰)	2.86 ± 0.71	2.45 ± 0.63	2.79 ± 0.37	2.57 ± 0.57
	PT (s)	16.52 ± 0.82	16.00 ± 1.20	16.27 ± 0.63	16.57 ± 1.29

∗Means *P* < 0.05; ∗∗means *P* < 0.01; *n* means the number of animals.

**Table 5:** The effects on blood biochemical and electrolyte indicators of MLE in SD rats ($\overset{-}{X}\pm \text{SD}$, g).

Sex	Parameters	Control group (*n* = 6)	Low-dose group (*n* = 6)	Medium-dose group (*n* = 6)	High-dose group (*n* = 6)
♂	ALT (U/L)	33.85 ± 5.16	40.90 ± 12.38	34.50 ± 4.71	34.82 ± 3.32
	AST (U/L)	143.57 ± 32.64	164.42 ± 49.16	144.92 ± 15.15	152.03 ± 48.87
	ALP (U/L)	83.73 ± 11.32	91.22 ± 30.39	83.65 ± 18.66	82.45 ± 17.09
	BUN (mmol/L)	6.22 ± 0.64	6.35 ± 0.73	6.72 ± 0.60	6.12 ± 0.57
	CREA (*μ*mol/L)	73.67 ± 7.55	70.35 ± 3.17	76.50 ± 3.31	72.05 ± 5.29
	TP (g/L)	57.53 ± 4.49	59.52 ± 1.09	59.22 ± 1.26	59.87 ± 3.34
	ALB (g/L)	22.90 ± 1.28	23.43 ± 0.74	23.67 ± 0.74	24.25 ± 0.90
	GLU (mmol/L)	10.14 ± 1.07	8.93 ± 1.13	10.35 ± 1.88	9.25 ± 0.93
	CHOl (mmol/L)	1.36 ± 0.26	1.78 ± 0.38	1.50 ± 0.39	1.74 ± 0.23*
	TBIL (*μ*mol/L)	2.34 ± 0.16	2.44 ± 0.28	2.39 ± 0.24	2.35 ± 0.15
	TG (mmol/L)	0.41 ± 0.16	0.48 ± 0.17	0.56 ± 0.28	0.64 ± 0.18*
	CK (U/L)	775.18 ± 354.51	812.22 ± 295.98	817.42 ± 143.23	795.83 ± 221.83
	Na^+^ (mmol/L)	139.62 ± 2.32	140.77 ± 2.27	139.65 ± 1.74	139.88 ± 0.37
	K^+^ (mmol/L)	4.52 ± 0.45	4.73 ± 0.19	4.58 ± 0.25	4.65 ± 0.30
	Cl^−^ (mmol/L)	104.00 ± 1.37	104.12 ± 1.40	102.90 ± 1.88	103.30 ± 1.11

♀	ALT (U/L)	26.35 ± 3.31	29.80 ± 4.60	25.98 ± 3.80	24.93 ± 4.54
	AST (U/L)	137.12 ± 18.95	128.62 ± 21.23	129.70 ± 23.13	114.12 ± 14.57*
	ALP (U/L)	42.28 ± 9.34	48.72 ± 13.80	43.47 ± 6.99	47.52 ± 10.20
	BUN (mmol/L)	7.67 ± 0.64	7.84 ± 0.74	7.56 ± 0.99	6.87 ± 1.31
	CREA (*μ*mol/L)	74.63 ± 4.22	76.52 ± 4.24	79.57 ± 4.44	73.15 ± 4.37
	TP (g/L)	65.97 ± 3.99	67.60 ± 3.47	67.98 ± 6.41	65.13 ± 3.23
	ALB (g/L)	29.37 ± 2.71	30.25 ± 1.94	30.57 ± 2.82	28.45 ± 2.26
	GLU (mmol/L)	7.96 ± 1.08	8.92 ± 1.00	8.93 ± 1.41	8.76 ± 1.00
	CHOl (mmol/L)	1.72 ± 0.19	1.79 ± 0.50	1.79 ± 0.43	1.63 ± 0.20
	TBIL (*μ*mol/L)	2.69 ± 0.20	2.68 ± 0.15	2.76 ± 0.43	2.42 ± 0.21*
	TG (mmol/L)	0.32 ± 0.06	0.34 ± 0.06	0.30 ± 0.06	0.31 ± 0.07
	CK (U/L)	607.28 ± 213.08	568.75 ± 167.69	670.88 ± 153.16	612.25 ± 136.05
	Na^+^ (mmol/L)	139.87 ± 1.30	140.55 ± 1.07	140.87 ± 1.24	139.68 ± 0.92
	K^+^ (mmol/L)	4.35 ± 0.31	4.18 ± 0.34	3.98 ± 0.23*	4.04 ± 0.32
	Cl^−^ (mmol/L)	104.92 ± 2.49	104.95 ± 0.36	104.82 ± 1.21	103.82 ± 0.60

∗Means *P* < 0.05; *n* means the number of animals.

**Table 6:** The effects on organ weights and the relative organ weights of MLE in male SD rats ($\overset{-}{X}\pm \text{SD}$, g).

Parameters	Control group (*n* = 6)	Low dose group (*n* = 6)	Medium dose group (*n* = 6)	High dose group (*n* = 6)
Organ weight (g)				
Heart	1.720 ± 0.311	1.715 ± 0.312	1.669 ± 0.127	1.640 ± 0.189
Liver	12.370 ± 1.468	12.673 ± 2.428	12.803 ± 2.070	13.091 ± 2.106
Spleen	0.783 ± 0.099	0.847 ± 0.121	0.788 ± 0.053	0.816 ± 0.124
Lung	1.623 ± 0.070	1.666 ± 0.215	1.711 ± 0.163	1.701 ± 0.147
Kidney	3.229 ± 0.418	3.040 ± 0.300	3.090 ± 0.318	3.207 ± 0.450
Brain	2.024 ± 0.057	2.138 ± 0.113	2.120 ± 0.123	2.058 ± 0.074
Adrenal gland	0.063 ± 0.010	0.062 ± 0.013	0.070 ± 0.008	0.070 ± 0.006
Thymus	0.397 ± 0.106	0.317 ± 0.064	0.396 ± 0.087	0.347 ± 0.038
Testis	3.348 ± 0.302	3.587 ± 0.293	3.529 ± 0.367	3.571 ± 0.320
Epididymis	1.557 ± 0.096	1.700 ± 0.121*	1.647 ± 0.208	1.787 ± 0.135**
Fat	14.949 ± 3.320	17.607 ± 5.071	18.648 ± 8.385	17.647 ± 4.952
Organ/body weight ratio (mg/g)				
Weight	514.2 ± 41.1	514.8 ± 55.9	520.0 ± 51.1	525.7 ± 59.0
Heart	3.335 ± 0.440	3.356 ± 0.696	3.217 ± 0.121	3.125 ± 0.218
Liver	24.012 ± 1.433	24.430 ± 2.333	24.504 ± 1.484	24.823 ± 1.562
Spleen	1.519 ± 0.097	1.640 ± 0.101	1.521 ± 0.115	1.552 ± 0.158
Lung	3.166 ± 0.174	3.233 ± 0.199	3.312 ± 0.406	3.249 ± 0.244
Kidney	6.269 ± 0.502	5.924 ± 0.453	5.974 ± 0.718	6.095 ± 0.424
Brain	3.953 ± 0.257	4.201 ± 0.587	4.114 ± 0.504	3.950 ± 0.406
Adrenal gland	0.124 ± 0.021	0.121 ± 0.022	0.135 ± 0.016	0.133 ± 0.017
Thymus	0.765 ± 0.164	0.622 ± 0.163	0.763 ± 0.176	0.664 ± 0.086
Testis	6.571 ± 1.005	7.010 ± 0.657	6.840 ± 0.972	6.848 ± 0.845
Epididymis	3.048 ± 0.349	3.317 ± 0.210	3.188 ± 0.472	3.426 ± 0.365
Fat	28.878 ± 4.757	33.652 ± 6.862	34.940 ± 11.830	33.614 ± 8.958
Organ/brain weight ratio (g/g)				
Brain	2.024 ± 0.057	2.138 ± 0.113	2.120 ± 0.123	2.058 ± 0.074
Heart	0.848 ± 0.140	0.810 ± 0.184	0.791 ± 0.090	0.797 ± 0.084
Liver	6.100 ± 0.585	5.955 ± 1.240	6.083 ± 1.263	6.365 ± 1.017
Spleen	0.386 ± 0.040	0.398 ± 0.065	0.373 ± 0.033	0.396 ± 0.057
Lung	0.802 ± 0.031	0.781 ± 0.112	0.810 ± 0.097	0.826 ± 0.062
Kidney	1.592 ± 0.172	1.428 ± 0.179	1.458 ± 0.123	1.556 ± 0.187
Adrenal gland	0.031 ± 0.005	0.029 ± 0.007	0.033 ± 0.004	0.034 ± 0.003
Thymus	0.196 ± 0.050	0.147 ± 0.024	0.188 ± 0.046	0.168 ± 0.017
Testis	1.656 ± 0.166	1.679 ± 0.123	1.669 ± 0.189	1.734 ± 0.119
Epididymis	0.770 ± 0.057	0.798 ± 0.080	0.781 ± 0.125	0.869 ± 0.063*
Fat	7.356 ± 1.451	8.266 ± 2.462	8.949 ± 4.533	8.578 ± 2.372

∗Means *P* < 0.05; ∗∗means *P* < 0.01; *n* means the number of animals.

**Table 7:** The effects on organ weights and the relative organ weights of MLE in female SD rats ($\overset{-}{X}\pm \text{SD}$, g).

Parameters	Control group (*n* = 6)	Low dose group (*n* = 6)	Medium dose group (*n* = 6)	High dose group (*n* = 6)
Organ weight (g)				
Heart	1.074 ± 0.162	1.059 ± 0.054	1.058 ± 0.100	1.092 ± 0.097
Liver	6.629 ± 0.449	7.320 ± 1.076	7.342 ± 0.747	7.215 ± 1.015
Spleen	0.484 ± 0.030	0.514 ± 0.058	0.470 ± 0.049	0.496 ± 0.073
Lung	1.230 ± 0.098	1.223 ± 0.084	1.256 ± 0.064	1.280 ± 0.116
kidney	1.751 ± 0.152	1.832 ± 0.169	1.875 ± 0.134	2.014 ± 0.227*
Brain	1.911 ± 0.054	1.905 ± 0.113	1.869 ± 0.084	1.905 ± 0.082
Adrenal gland	0.064 ± 0.013	0.082 ± 0.017	0.070 ± 0.012	0.084 ± 0.013*
Thymus	0.266 ± 0.052	0.282 ± 0.074	0.308 ± 0.065	0.264 ± 0.096
Uterus	0.726 ± 0.156	0.733 ± 0.107	0.756 ± 0.107	0.641 ± 0.184
Ovary	0.186 ± 0.025	0.194 ± 0.030	0.174 ± 0.036	0.195 ± 0.028
Fat	8.844 ± 2.096	9.452 ± 2.210	10.023 ± 4.202	8.612 ± 4.182
Organ/body weight ratio (mg/g)				
Weight	283.2 ± 13.8	286.5 ± 14.2	289.0 ± 26.4	291.8 ± 35.2
Heart	3.791 ± 0.516	3.705 ± 0.283	3.666 ± 0.244	3.770 ± 0.440
Liver	23.404 ± 0.952	25.490 ± 2.803	25.445 ± 1.930*	24.696 ± 1.327
Spleen	1.709 ± 0.079	1.793 ± 0.171	1.630 ± 0.130	1.702 ± 0.146
Lung	4.341 ± 0.244	4.267 ± 0.190	4.364 ± 0.286	4.405 ± 0.269
kidney	6.190 ± 0.561	6.388 ± 0.388	6.503 ± 0.327	6.919 ± 0.411*
Brain	6.757 ± 0.252	6.667 ± 0.568	6.509 ± 0.622	6.608 ± 0.872
Adrenal gland	0.226 ± 0.043	0.286 ± 0.050	0.242 ± 0.035	0.294 ± 0.068
Thymus	0.937 ± 0.163	0.986 ± 0.254	1.064 ± 0.197	0.900 ± 0.299
Uterus	2.567 ± 0.557	2.563 ± 0.379	2.636 ± 0.438	2.190 ± 0.518
Ovary	0.659 ± 0.102	0.682 ± 0.137	0.608 ± 0.153	0.674 ± 0.095
Fat	31.288 ± 7.420	32.888 ± 6.624	33.915 ± 11.504	28.450 ± 11.684
Organ/brain weight ratio (g/g)				
Brain	1.911 ± 0.054	1.905 ± 0.113	1.869 ± 0.084	1.905 ± 0.082
Heart	0.562 ± 0.081	0.559 ± 0.063	0.567 ± 0.060	0.573 ± 0.047
Liver	3.468 ± 0.195	3.857 ± 0.622	3.926 ± 0.318*	3.790 ± 0.526
Spleen	0.253 ± 0.011	0.270 ± 0.029	0.252 ± 0.026	0.261 ± 0.042
Lung	0.643 ± 0.050	0.643 ± 0.050	0.672 ± 0.023	0.673 ± 0.063
kidney	0.916 ± 0.075	0.966 ± 0.119	1.005 ± 0.084	1.057 ± 0.103*
Brain	0.033 ± 0.006	0.043 ± 0.010	0.037 ± 0.006	0.044 ± 0.005**
Adrenal gland	0.139 ± 0.024	0.149 ± 0.041	0.165 ± 0.033	0.139 ± 0.053
Thymus	0.380 ± 0.082	0.385 ± 0.049	0.406 ± 0.062	0.338 ± 0.104
Uterus	0.098 ± 0.014	0.102 ± 0.018	0.094 ± 0.022	0.103 ± 0.016
Ovary	4.645 ± 1.166	4.979 ± 1.183	5.390 ± 2.372	4.525 ± 2.194

∗Means *P* < 0.05; ∗∗means *P* < 0.01; *n* means the number of animals.
